# Regulations of T Cell Activation by Membrane and Cytoskeleton

**DOI:** 10.3390/membranes10120443

**Published:** 2020-12-19

**Authors:** Yoshihisa Kaizuka

**Affiliations:** National Institute for Materials Science, Tsukuba, Ibaraki 305-0047, Japan; KAIZUKA.Yoshihisa@nims.go.jp

**Keywords:** actin, immunological synapse, catch bond, agonist, MHC, LAT, CD45, mechanical force

## Abstract

Among various types of membrane proteins that are regulated by cytoskeleton, the T cell receptor (TCR) greatly benefits from these cellular machineries for its function. The T cell is activated by the ligation of TCR to its target agonist peptide. However, the binding affinity of the two is not very strong, while the T cell needs to discriminate agonist from many nonagonist peptides. Moreover, the strength and duration of the activation signaling need to be tuned for immunological functions. Many years of investigations revealed that dynamic acto-myosin cytoskeletons and plasma membranes in T cells facilitate such regulations by modulating the spatiotemporal distributions of proteins in plasma membranes and by applying mechanical loads on proteins. In these processes, protein dynamics in multiple scales are involved, ranging from collective molecular motions and macroscopic molecular organizations at the cell–cell interface to microscopic changes in distances between receptor and ligand molecules. In this review, details of how cytoskeletons and membranes regulate these processes are discussed, with the emphasis on how all these processes are coordinated to occur within a single cell system.

## 1. Introduction

The T cell receptor (TCR) is the immune receptor expressed in T cell plasma membranes and recognizes a short antigenic peptide bound to the major histocompatibility complex (MHC) that is expressed on antigen-presenting cells (APCs) [1]. This binding triggers T cell activation, resulting in a wide range of immune functions depending on the stages of T cell development and differentiation, although intracellular molecular systems to regulate activation processes in T cells are largely common in different situations and have been well-studied at the molecular and cellular levels.

Among such intracellular molecular machinery, the acto-myosin cytoskeleton has been long recognized as a critical factor in T cell activation and TCR triggering [2,3,4,5,6]. First, the study using the pharmacological inhibition of actin polymerization revealed its crucial role in the very early TCR-triggering process by APCs [7]. The loss of the Wiskott-Aldrich syndrome protein (WASp), a direct activator of the Arp2/3 complex for actin nucleation and an important molecule in the TCR signaling pathway, results in severely impaired cellular and humoral immunity, confirming the importance of the actin cytoskeleton in T cell immunity [8,9]. The plasma membrane is also a key structure to understand the functions of the T cell. The TCR and its proximal signal molecules, as well as important coreceptors, function in the confined space between two opposed plasma membranes of the T cell and APC. The acto-myosin cytoskeleton and plasma membrane are structurally and functionally coupled via membrane proteins that anchor the cytoskeleton or elicit actin polymerization. Indeed, a variety of interesting cellular processes that could be regulated by the interplay of dynamic acto-myosin cytoskeleton and plasma membrane structures have been observed.

**Figure 1:**
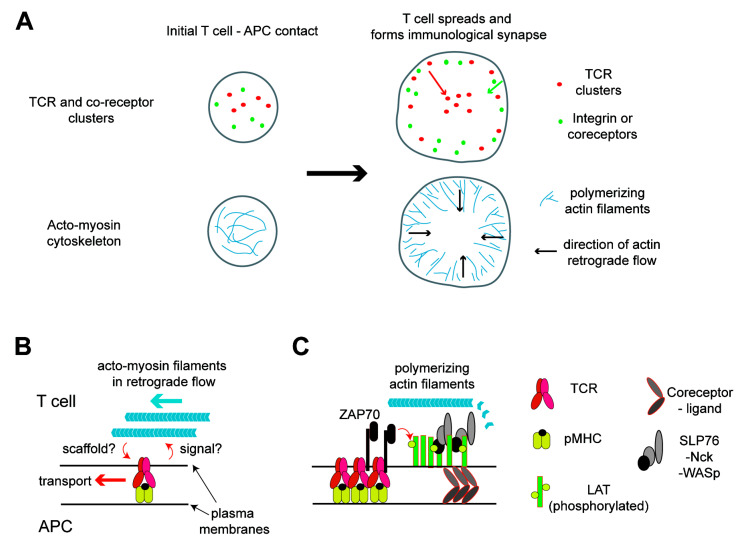
Acto-myosin cytoskeleton network in activated T cells and its roles in signaling and molecular transport. (**A**) Distribution of the T cell receptor (TCR) and actin at the interface between the T cell and antigen-presenting cell (APC). At the initial contact of the two cells, the TCR and coreceptors nucleate the clusters by binding to their ligands and cortical actin nucleates. This active actin polymerization induces the spreading of the T cell over the APC soon after, resulting in the formation of a lamella structure at the periphery. Polymerized acto-myosin filaments exhibit a retrograde flow in the lamella. These filaments function as scaffolds or induce the transport of protein clusters toward the cell center. The TCR moves a longer distance to the center, while the coreceptors and integrin remain in the periphery, resulting in a bull’s eye pattern of the immunological synapse. (**B**) TCR proximal signaling leads to the nucleation of actin filaments, which, in turn, function as scaffolding in the protein cluster transport. (**C**) Details in the link between the TCR and actin. ZAP70 recruited to the phosphorylated immunoreceptor tyrosine-based activation motifs (ITAMs) in the TCR-CD3 complexes phosphorylates tyrosine residues in the linker for the activation of T cells (LAT). Then, signalsome proteins, such as SLP76, Nck, and WASp, are recruited to the phosphorylated LAT to form molecular condensation, which is likely colocalized with the TCR at least for a short period of time and can also be colocalized with coreceptor domains. These protein clusters can nucleate actin filaments, which can scaffold the clusters temporarily. pMHC: peptide major histocompatibility complex.

**Figure 2:**
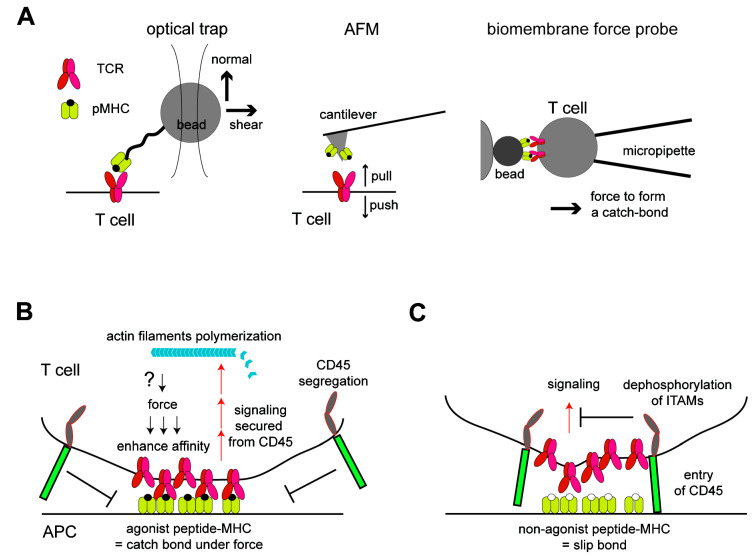
Mechanical regulations of TCR activation. (**A**) Biophysical assays to probe the mechanical regulations of TCR. Either an optical trap, atomic force microscope (AFM), or biomembrane force probe can load and measure the force in the binding between the TCR and pMHC. T cell activation can be measured simultaneously by imaging the calcium flux. (**B**) Roles of force in T cell triggering induced by ligation to the agonist peptide MHC. Force generated at the membrane interface enhances the affinity between TCR and pMHC by forming a catch bond, resulting in the formation of a tight membrane–membrane junction. These junctions spatially exclude CD45 by blocking the entry of the bulky and highly glycosylated extracellular domain of CD45 in this confined space. In this geometry, the signaling stemming from phosphorylated ITAMs in the TCR can be more active by securing it from the tyrosine dephosphorylation by CD45. The signaling leads to local actin nucleation, which likely functions to load forces on the TCR. (**C**) Such a feedback loop is not generated in the binding between the TCR and nonagonist peptide MHC, which only forms a slip bond. The binding of these two molecules remains a lower affinity, and the junction between these cells cannot be tight enough to exclude CD45. The entry of CD45 blocks the signaling from the TCR to downstream by dephosphorylating ITAMs in the TCR.

In this review, after a brief introductory section about basics of T cell signaling, I will discuss how the interplay of the membrane and acto-myosin cytoskeleton in T cells regulate T cell activation. First, the roles of acto-myosin in the formation of spatial organizations of membrane proteins and the immunological synapse are discussed. During T cell activation, the TCR and other signaling molecules in T cell plasma membranes were found not only to interact with but also to form submicron-to-macroscopic-sized organizations [10,11,12,13,14]. How T cells generate the dynamic actin cytoskeleton and how the cytoskeleton coordinates these collective molecular motions in membranes are discussed (Figure 1). In the second section, the roles of the mechanical forces in TCR triggering are discussed. Many recent investigations have suggested that mechanical loads are important in T cell signaling [4,6,15]. Particularly, it is demonstrated that the TCR molecule can sense and transduce force. Thus, the focus in this section is microscopic molecular dynamics, although interestingly, the macroscopic membrane and cytoskeleton structures are also very important (Figure 2). Altogether, it illustrates how the membrane and cytoskeleton are coordinated to regulate a wide range of protein organizations and dynamics in all lengths of scales to achieve one task: T cell triggering. Intensive investigations have made T cells an interesting platform to study such interplays of the membrane and cytoskeleton in signal transduction, where we can take advantage of a large amount of biochemical information and many experimental tools available.

## 2. Mechanisms of TCR-Triggering and Downstream Signaling

The TCR is a complex of the αß TCR heterodimer that recognizes the antigenic peptide and CD3 polypeptides to transduce signaling downstream through multiple phosphorylation sites (the immunoreceptor tyrosine-based activation motif (ITAM) sequence) [16]. The TCR transduces signals downstream as other well-studied receptors do, while there are some unique features, including actin-based regulations and complex roles of phosphatase CD45, in signal regulations, as discussed later [16,17]. In contrast, the processes of ligation and triggering of the TCR are different from the binding of conventional receptors and their ligands [18,19]. The expression of the TCR in T cells is not low, 10^4^~10^5^ molecules per cell [20], but the expression of antigenic peptide MHCs in APCs are sometimes at a low copy number, while irreverent MHC molecules bound with self-peptides are highly expressed. Thus, the TCR requires very sensitive and specific binding to its cognate peptide MHC, although their binding affinity in solution is not high and the dissociation constants in the micro-molar range or higher. To explain how the T cell searches its specific cognate peptide with a low copy number and low affinity in vivo, the concepts of “kinetic proofreading” and/or “serial triggering” were introduced [21,22]. In the kinetic proofreading model, the TCR molecules scan vast amounts of peptide MHC molecules to find the cognate peptide at the interface between the T cell and APC, where relatively high dissociation constants in the binding help such a proofreading process. The serial triggering model was introduced to explain how T cell signaling can be sustained for long enough to fully activate T cells for gene upregulations (hours, in contrary to a few seconds of the lifetime of the TCR and peptide MHC binding). In this model, many TCRs are triggered serially by a small number of peptide MHC, while they also do kinetic proofreading. This model also explains the downregulation of TCR molecules that are once triggered, which was used as a measure of TCR triggering [18]. Such an internalization of the TCR is an important molecular transport event in regarding T cells and is still actively investigated [23].

TCR-triggering leads to the phosphorylation of tyrosine residues in ITAMs by the proximal Src family kinase Lck. Another tyrosine kinase, ZAP70, is then recruited to the phosphorylated ITAM domains and elicits downstream signaling by phosphorylating a key adaptor molecule, the linker for the activation of T cells LAT [16,17]. LAT contains multiple tyrosine residues to be phosphorylated by ZAP70 that are used for interactions with downstream proteins. Actin nucleation is one of the key events downstream of LAT besides the intracellular calcium release or Ras activation. WASp (or, also, WAVE2) is involved in this actin nucleation process in T cells by activating the Arp2/3 complex, and an important finding was that LAT formed protein clusters colocalized with WASp and Nck (an adaptor for WASp to interact with LAT) and nucleated actin [10,24]. Another key regulator of TCR proximal signaling is phosphatase CD45 [16]. Interestingly, CD45 is essential for TCR-triggering, and this positive regulation is mediated by releasing the autoinhibitory interaction of Lck via dephosphorylating the tyrosine near the C-terminus end to activate Lck. However, CD45 is highly expressed in the T cell membrane and has a significant impact on TCR phosphorylation. “Kinetic segregation”, another model for TCR-triggering that includes downstream kinase and phosphatase molecules as regulators, is based on the idea that the spatial exclusion of CD45 from TCR–pMHC can secure phosphorylated TCR from dephosphorylation by CD45 and help the induction of downstream signaling [25,26,27]. This exclusion is induced by the steric mismatch in the confined membrane–membrane interface, because the extracellular domain of CD45 (>20 nm) is bulky and highly glycosylated and is larger than the distance of TCR–pMHC binding (~10 nm), and the model is compatible with observations of domain and cluster formations in T cell membranes. It is also well-studied that the affinity between the TCR and pMHC is effectively enhanced as they are confined in the space between the T cell and APC [28]. Coreceptors are also important regulators in T cell signaling. At the interface between T cells and APCs, not only typical adhesion proteins such as integrins but, also, coreceptor proteins that can harbor signaling other than TCR conjugate to their ligands. CD28 or ICOS are stimulatory coreceptors that can reduce the threshold of the number of triggering TCR for T cell activation or be useful for avoiding an anergy state [29,30,31], while PD-1, CTLA-4, or LAG-3 are inhibitory coreceptors and are critical in immune checkpoint therapies [32]. CD2 may have both stimulatory and adhesive functions [33]. These coreceptors modulate signaling by sharing signal proteins and the actin cytoskeleton with the TCR.

## 3. Roles of Acto-Myosin Cytoskeleton in the Formation of Protein Organizations in T Cell Membranes

### 3.1. Protein Organizations in Plasma Membranes of Activated T Cells

T cells form spatial organizations of proteins in plasma membranes, and the acto-myosin cytoskeleton is involved in this process. These protein organizations have two distinct scales: submicron protein clusters and macroscopic pattern of these clusters or the immunological synapse. The former is the unit for signaling reactions and is most likely formed by protein–protein interactions. In contrast, the macroscopic pattern of the immunological synapse is a hallmark of initial T cell activation reactions, while the immunological function of patterning is currently unknown. The roles of actin in these protein organizations were first suggested from studies using pharmacological inhibitors, and recent investigations are focused on the mechanisms, as discussed in this section.

The immunological synapse, a bull’s eye pattern of the TCR and adhesion molecules formed in the membrane interface between the T cells and APCs was later resolved as the accumulation of many smaller-sized protein clusters [12,13,14,34]. These analyses were enabled in higher resolutions by using ligands anchored to artificial lipid bilayers as mimics of APC surfaces, combined with various imaging techniques ranging from conventional total internal reflection microscopy to super-resolution microscopies, as well as with surface fabrication technologies [10,11,12,27,34,35,36,37,38]. Nucleated protein clusters are enriched with TCR and signaling molecules, and they are highly active in the tyrosine phosphorylation signaling of ITAMs. The bull’s eye pattern of the immunological synapse is formed by accumulation of these nucleated domains transported toward the center of the cell–cell interface (Figure 1A). However, interestingly, the phosphorylation signaling in domains, as well as the colocalizations of other signal proteins, are diminished, while the domains are transported to the center [12,27,34,35,39]. Besides the accumulation of signal proteins and amplification of the reactions, one major role of protein clustering is the spatial exclusion of CD45 from the TCR [25,26]. Other types of signal modulations in protein condensation proposed previously include the modulation of Lck activity by balancing the negative regulation (Lck autophosphorylation) and positive regulation (dephosphorylation by CD45) through the control of the protein mobility or the control of the membrane retention time for Son of Sevenless (SOS) to facilitate kinetic proofreading to modulate the Ras activation [40,41].

Along with the formation of the immunological synapse, the inhibition of actin filament polymerization abrogates all of the nucleation, phosphorylation, and transport of the TCR domains [27,35]. In addition to the TCR and integrin LFA-1 conjugated with ICAM-1, other coreceptors (CD28, CTLA-4, CD2, and PD-1) conjugated with their ligands also form domains in membranes and play roles in modulating their signals [42,43,44,45,46]. These coreceptor domains are not only segregated from each other and from the TCR but, also, are distributed radially throughout the membrane interface. Moreover, the coreceptor domains were also regulated at least in part by the actin cytoskeleton [42,43,44,46,47].

### 3.2. Dynamics of the Acto-Myosin Cytoskeleton in T Cell Membranes and Its Role in Protein Organizations

The roles of the actin cytoskeleton in the immunological synapse were investigated by imaging (Figure 1A,B). Earlier studies showed that the TCR cluster formation and signaling induced a lamella formation at the cell periphery through active actin polymerization, resulting in the spreading of T cells [27,35]. As mentioned above, ligated TCR molecules form domains at the periphery and are transported to the center, resulting in the structure called cSMAC (central supramolecular activation cluster). In contrast, LFA-1 forms clusters at the cell periphery, and coreceptors (CD28, PD-1, or CD2) are located in the space between the cSMAC and periphery [3,13]. Thus, the lamella formation indicates not only that signal proteins for actin polymerizations are located at the cell periphery but, also, that these lamellae may regulate the localizations of LFA-1 and other coreceptors in the cell periphery or motions of these protein domains. Indeed, the simultaneous imaging of the TCR, actin, and LFA-1 suggest that the protein domains (both the TCR and LFA-1 and others) are transported with actin filaments in retrograde flow, and the localization of the LFA-1 domain is limited to the actin-rich lamella area, resulting in a bull’s eye pattern [44,47]. The actin and myosin-based transport of the protein domains were further investigated and confirmed by the physical modulation of the domain transport with a fabricated surface and by the super-resolution imaging of the cytoskeleton architecture [6,48,49,50]. These studies revealed molecular organizations of actin filaments and related proteins at the interface of T cells and APCs in great detail, resulting in a better understanding of cytoskeleton-based regulations of molecular organizations in plasma membranes [6].

The acto-myosin cytoskeleton dynamics and domain transport and partitioning within the T cell immunological synapse are intriguing phenomena. While they reflect important signaling events and is observed both in vitro and in vivo, it is still unclear how these dynamic phenomena are related to immunological functions. Meanwhile, investigations on signaling molecules that link the TCR to actin are progressing in the context of membrane domain formations (Figure 1C). LAT has been the key molecule in these studies. Colocalizations of LAT and downstream signaling proteins, including WASp, in activated T cell membranes were shown in earlier studies [10,24]. Then, a series of studies showed that LAT and the TCR are largely spatially segregated, or mixed only partially, either in resting cells or in cells activated by immobilized ligands or by APC-mimicked lipid bilayers [11,35,36,37,38,51]. The biochemical reconstitution of LAT and the downstream molecules showed that these proteins exhibit liquid–liquid-phase separations in a membrane-anchored format, and actin was nucleated in these phase-separated domains enriched with adaptor molecules (Grb2, SLP76, and Nck) and WASp [52,53,54]. Collectively, it is likely that TCR–pMHC ligates form domains that initiate LAT phosphorylation by recruited ZAP70 in the domains [12]. However, after a while, phosphorylated LAT condenses with other downstream signaling proteins separately from the TCR, as shown in T cells activated by immobilized TCR ligands. T cells may also use coreceptors to form domains enriched with LAT and downstream signaling proteins separately from TCR domains [36,37,42,44]. During this spatial transition of LAT and the downstream molecules, actin nucleation occurs, and molecular linkers that possibly consist of LAT-associated adaptor molecules and WASp may function as a scaffold in the transporting of protein domains toward the cell center. However, that linkage between TCR–LAT–actin may be lost after the separation of LAT from the TCR, resulting in a loss of signaling activity and accumulation of nonsignaling domains in the cell center. This model is still incomplete, and further analyses are required to understand the whole processes. In addition, these studies could also benefit from physical and mathematical modeling, which have greatly contributed to the T cell biology since the pioneering work by Groves and Chakraborty [55,56]. The T cell biology has attracted many physical scientists due to its interesting complexity that consists of not only biochemical signaling reactions but, also, dynamic membrane and cytoskeleton structures.

Electron microscopy imaging of the interface of the T cell and APCs revealed actin-rich pseudopodia structures in T cells that are penetrating APCs [57]. Further investigations using super-resolution imaging have revealed the roles of microvilli structures in the search for APCs [58] and the details of molecular organizations and signaling in these structures [59,60,61], which are, in part, regulated by actin filaments. Another interesting role of actin was observed in a cytotoxic T cell, where actin may have been used to enhance the membrane tension to induce the release of lytic granules at the immunological synapse [62]. Periphery actin polymerization has roles in cell migration as well that are essential for the T cell immunity of finding APCs in vivo. Additional molecular systems such as protein kinase C-θ (PKCθ) signaling may be involved in the initiation of migration through the destabilization of the immunological synapse [63], and myosin1 g has a role in meandering cells during migration for the efficient search of APCs [64].

## 4. Mechanical Regulation of T Cell Activation

### 4.1. Forces and TCR-Triggering

Recent studies showed that the αß TCR molecule may sense forces and may be regulated mechanically [15]. As the generation of acto-myosin cytoskeleton dynamics is quite active at the interface between T cells and APCs, as discussed in the previous section, thus, the triggering of TCR at the cell–cell interface may be mechanically regulated by actin cytoskeleton. Various types of quantitative analyses, as well as models for the mechanisms in the mechanical regulation of T cell activation, are introduced in this section.

Among various biophysical tools to mechanically manipulate T cells or TCR molecules, optical traps and micropipette aspirations have been applied in earlier studies (Figure 2A). In these assays, either a single cell that mimics APC or a bead coated with peptide MCH was trapped and manipulated by optical tweezers or a glass micropipette [15,65,66]. Trapped APCs or ligand-coated beads were once interacted with T cells, and then, they were moved toward either a normal or tangent direction, and T cell activation was monitored by the calcium flux. In early studies, Kim et al. showed that the shear force driven by tangential motion is important for activation, while Li et al. showed that forces in both the normal and tangential directions can activate T cells [65,66]. The direction of applied forces to the TCR in these experiments are important, because it reflects how the force can be applied in cells and will be discussed in more detail in a later section. These studies demonstrated the utility of biophysical tools to study this subject, and the study by Li et al. also suggested that this mechanical transduction of T cell activation occurred in cell–cell interfaces. Following these initial studies, a careful analysis by using optical tweezers demonstrated the thresholds of a number of peptide MHCs and forces to activate T cells, as there were 2~29 molecules per T cell–bead interface at the loaded force of 8~25 pN [67]. These ligand density thresholds are a few orders of magnitude lower than those required to activate T cells in the absence of force, ~2 × 10^4^ molecules in the interface, suggesting a significant role of force in T cell triggering [67]. Furthermore, when the pulled beads in shear direction to trigger T cells were relaxed and back at the original position, the beads exhibited motions with discrete step sizes that are characteristics of myosin motor-dependent transport. These steps disappeared by adding inhibitors for actin polymerization and myosin function. Taken together, acto-myosin filaments may be involved in the force generation to trigger TCR. Note that the force in the normal direction was also probed to activate T cells in similar ranges of forces and ligand densities in the same assay. An atomic force microscope (AFM) was also employed for studying the TCR mechanical regulation by using the peptide MHC-decorated AFM cantilever as both the force actuator and force probe. The study using AFM showed that activated T cells were shown to push and pull the ligand-coated surfaces, indicating a force generation by activated TCR in the normal direction [68]. The inhibitors for the acto-myosin cytoskeleton abrogated the T cell activation and motions in activated T cells, and interestingly, T cell activation was rescued by applying pull-push cyclic forces by AFM even in the absence of acto-myosin filaments. Sophisticated fluorescence-based measurements were also developed using a DNA-anchored ligand fabricated on the surface. In this assay, the molecular tension from one side was converted to the gain of fluorescence that was initially quenched, and it measured a similar range of force, 12~19 pN, transmitted through the binding of the TCR and pMHC [69]. Traction force microscopy (TFM) using an engineered substrate also enabled the force measurements [70,71,72]. In these TFM assays, T cells interacted with ligands immobilized on elastic substrate surfaces or flexible polymer pillars fabricated on the surface, and the forces transmitted between the T cells and substrates through the ligated TCRs were measured as changes in the positions of the fluorescent beads embedded in the substrate or as a deflection of the pillars. TFM is useful for measuring forces in a tangential direction in a high spatial and temporal resolution and showed a potential adjustment of the speed of actin retrograde flow by the force from the TCR [71].

These biophysical characterizations led to analyses of how TCRs respond or transduce forces in molecular levels. Among these analyses, the TCR was shown to mechanically discriminate its ligands. This is first done by using a biomembrane force probe (BFP), an assay with two micropipettes, where one pipette holds and pulls a T cell with force and the other pipette holds a model APC or a ligand-coated bead conjugated to the surface of a red blood cell [73] (Figure 2A). When the TCR binds to a potent agonist peptide MHC that can trigger T cells, the lifetime of binding was prolonged in the middle range of the pulling force, ~10 pN, while such an extension of the lifetime was not observed for lower or higher forces. Such a biphasic manner of molecular binding is called a catch-bond and was shown previously for other molecules [74,75,76]. In contrast, in the bindings between the TCRs and nonagonists that showed a similar dissociation constant but did not elicit T cell activation, the increasing of the pulling forces monotonically enhanced the chance of a bond break (called a “slip bond”). These results show that the binding affinity between the TCR and agonist peptide can be enhanced under the force, but that is not applied to the binding with the nonagonist. These distinct binding modes of TCRs with agonists and nonagonists were also observed at the single-molecule level by the optical trap assay [77]. Other assays also revealed differential responses of the TCR to agonists and nonagonists in a variety of settings [69,71]. Notably, molecular structures of the TCR–pMHC-binding interface were also interpreted in the context of the catch bond and slip bond by a molecular dynamics simulation [78,79,80]. These simulation results indicated that applied forces could stabilize the binding by modulating the relative conformations and increasing the number of hydrogen bonds at the interface during a catch bond formation of TCR–pMHC, although such conformational changes have not been resolved by experimental analyses. Another important observation at a larger-length scale was that the exclusion of phosphatase CD45 from the TCR was also different between the ligations of the TCR with an agonist or antagonist. The exclusion of CD45 occurred only when the TCR binds to an agonist [13,78,81,82]. The exclusion of CD45 determines the signaling outcome by modulating the degree of ITAM phosphorylation, and thus, this can be a critical mechanism to discriminate agonist against antagonist. Mechanistically, the spatial exclusion of CD45 is driven by the local accumulation of TCR–pMHC bonds at the membrane interface [25]. The discrimination of the catch bond and slip bond may underlie the differential kinetics in the bond formation [83]; thus, it may result in differential outcomes of the lateral exclusion of CD45. Altogether, these results suggest that the binding of TCR and the agonist peptide–MHC senses a certain force, resulting in prolonged binding lifetime and enhancing of the signaling strength by altering the lateral membrane protein organizations (Figure 2B,C).

### 4.2. Acto-Myosin Cytoskeleton and TCR-Triggering

These series of studies on the mechanical regulations in T cell activation indicate that force is critical. How the force is generated and applied is not understood well, but the acto-myosin cytoskeleton is likely involved, which could explain why the actin cytoskeleton is crucial in T cell triggering. To understand how the force is generated, the direction of the force can be important. As discussed in the previous section, both the shear and normal forces could trigger the signals. The shear force can be generated by actin polymerization in a retrograde flow that is parallel to the plasma membrane surface, while the normal force can be generated by microvilli and pseudopodia that are also modulated by actin filaments. The shear force can also be generated by the macroscopic cell motion or external flow. As also discussed earlier, the linkage between the TCR and actin is not fully understood but may involve molecules in the LAT signalsome via the membrane domains. In either direction in force generation, there should be a feedback mechanism: TCR signaling creates a LAT signalsome and induces actin nucleation, which could function as a scaffold to generate and transmit a force that is required for TCR-triggering. The plasma membrane is also the platform to facilitate all of these events, including the CD45 exclusion that may function to trigger and tune the signaling.

There are still many questions remaining. Catch bond formation may be a key system to explain how the TCR can find a small number of cognate ligands with relatively low affinity; while there are other receptor–ligand systems showing similar catch bond formations [74,75,76]. Thus, while the discrimination between the catch bond for the TCR agonist and slip bond for the TCR nonagonist may be important in T cell functions, the intracellular system to facilitate the catch bond formation is not special for T cells. Moreover, the force generation may also be a common regulation mechanism for various membrane receptors, and similar ~pN forces were measured for other receptors [84,85]. The regulations of membrane receptors by the actin cytoskeleton are also very common, and many direct or indirect mechanisms have been investigated in great detail [86,87,88,89]. Meanwhile, the catch bond formation was not observed in the binding of the TCR and agonist pMHC in a flow chamber-based assay of binding between ligands immobilized on the surface and receptor-coated microspheres [90]. These inconsistent results suggest that the force generation and signal triggering may be geometry-dependent, which is consistent with the well-known fact that T cell activation requires surface-bound ligands, multimerized ligands, or fluid membrane-anchored ligands [91,92]. Another factor related to the geometry is the mechanical property of the opposed surface, either an artificial substrate or APC. Recent studies have suggested that the stiffness of these opposed surfaces might regulate T cell activation [93,94].

## 5. Conclusions and Future Perspectives

Altogether, it is shown that T cell activation, or the regulation of membrane receptors at the interface consisting of a dynamic plasma membrane and cytoskeleton more generally, is a fascinating problem that links microscopic molecular conformation and macroscopic intracellular structures to execute biological functions. Either of the topics discussed in this text, the dynamics of the atco-myosin cytoskeleton or force-based regulation of the TCR, is just one face of the whole machinery of T cell activation. The whole system also includes the mechanisms of feedback and amplification based on protein organization and exclusion, which are all dynamic and plastic and are regulated not only by specific protein interactions but, also, by the interplay of the acto-myosin cytoskeleton and plasma membrane structure. More efforts from different perspectives should be combined to fully understand the whole process in the future.
